# Supplementary material for: A population genetic assessment of coral recovery on highly disturbed reefs of the Keppel Island archipelago in the southern Great Barrier Reef
Source: PeerJ. 2015 Jul 23;3:e1092. doi: 10.7717/peerj.1092 (PMC4517960; doi:10.7717/peerj.1092)
Supplement: Table S1 [file peerj-03-1092-s001.docx]

Table S1: The four triplex reactions, fluorescent labels, expected fragment sizes and repeat unit size for the twelve microsatellite loci used to identify genetic population structure of *Acropora millepora*.

| Reaction # | Label | Locus | Expected min allele size bp | max allele size bp | Repeated nucleotide |
| --- | --- | --- | --- | --- | --- |
| Rn 1 | HEX | Amil2 006 | 93 | 133 | 2 |
|  | TET | Amil2 022 | 161 | 179 | 2 |
|  | FAM | Amil2 002 | 93 | 101 | 2 |
|  |  |  |  |  |  |
| Rn 2 | FAM | Amil2 010 | 156 | 192 | 2 |
|  | HEX | Amil2 023 | 129 | 179 | 2 |
|  | TET | Amil2 008J | 168 | 198 | 2 |
|  |  |  |  |  |  |
| Rn 3 | HEX | WGS 189 | 158 | 194 | 4 |
|  | TET | WGS 035 | 162 | 188 | 4 |
|  | FAM | WGS 051 | 151 | 216 | 4 |
|  |  |  |  |  |  |
| Rn 4 | HEX | WGS 134 | 105 | 133 | 4 |
|  | TET | WGS 196 | 128 | 234 | 4 |
|  | FAM | WGS 152 | 87 | 110 | 2 |
