# Supplementary material for: A population genetic assessment of coral recovery on highly disturbed reefs of the Keppel Island archipelago in the southern Great Barrier Reef
Source: PeerJ. 2015 Jul 23;3:e1092. doi: 10.7717/peerj.1092 (PMC4517960; doi:10.7717/peerj.1092)
Supplement: Table S2 — N, number of samples per locus and population after removal of repeated MLGs; A, number of alleles; HE, expected heterozygosity; HO observed heterozygosity; FIS, inbreeding coefficient. Italic font indicates statistical significance after FDR correction. [file peerj-03-1092-s002.docx]

Table S2: Basic statistics for all loci in the nine Keppel Island populations for which new data were obtained in this study.N = number of samples per locus and population after removal of repeated MLGs, A = number of alleles, *H_E_* = expected heterozygosity, *H_O_* = observed heterozygosity, *F_IS_* = inbreeding coefficient. Italic font indicates statistical significance after FDR correction.

| **Population** |  |  |  |  |  |  |  |  |  |  |  |
| --- | --- | --- | --- | --- | --- | --- | --- | --- | --- | --- | --- |
|  | **Locus** | **Amil2_002** | **Amil2_006** | **Amil2_008** | **Amil2_010** | **Amil2_022** | **Amil2_023** | **WGS_035** | **WGS_134** | **WGS_189** | **WGS_196** |
| **Barren** | **N** | 29 | 29 | 25 | 29 | 29 | 29 | 22 | 28 | 29 | 29 |
| **Island** | **A** | 7 | 5 | 11 | 4 | 12 | 6 | 8 | 4 | 12 | 7 |
|  | ***H_O_*** | 0.345 | 0.172 | 0.600 | 0.345 | 0.828 | 0.517 | 0.727 | 0.214 | 0.862 | 0.207 |
|  | ***H_E_*** | 0.773 | 0.455 | 0.830 | 0.441 | 0.831 | 0.628 | 0.689 | 0.365 | 0.773 | 0.668 |
|  | ***F_IS_*** | *0.554* | *0.621* | *0.277* | 0.217 | 0.004 | 0.177 | -0.055 | *0.413* | *-0.115* | *0.690* |
| **Halftide** | **N** | 50 | 50 | 46 | 50 | 50 | 50 | 49 | 50 | 50 | 50 |
| **Rocks** | **A** | 6 | 5 | 3 | 8 | 9 | 5 | 9 | 6 | 15 | 12 |
|  | ***H_O_*** | 0.480 | 0.220 | 0.087 | 0.580 | 0.780 | 0.420 | 0.490 | 0.560 | 0.600 | 0.580 |
|  | ***H_E_*** | 0.477 | 0.513 | 0.232 | 0.690 | 0.671 | 0.391 | 0.713 | 0.683 | 0.779 | 0.759 |
|  | ***F_IS_*** | *-0.006* | *0.571* | *0.624* | *0.160* | -0.162 | -0.074 | *0.313* | 0.180 | *0.229* | *0.236* |
| **Halfway** | **N** | 50 | 50 | 48 | 50 | 50 | 50 | 48 | 50 | 50 | 50 |
| **Island** | **A** | 5 | 5 | 4 | 9 | 10 | 6 | 7 | 5 | 12 | 13 |
|  | ***H_O_*** | 0.620 | 0.240 | 0.167 | 0.660 | 0.700 | 0.740 | 0.438 | 0.500 | 0.740 | 0.480 |
|  | ***H_E_*** | 0.549 | 0.465 | 0.472 | 0.711 | 0.668 | 0.637 | 0.705 | 0.600 | 0.783 | 0.681 |
|  | ***F_IS_*** | -0.129 | *0.484* | *0.647* | 0.072 | -0.048 | -0.161 | *0.380* | 0.167 | 0.054 | *0.295* |
| **Humpy** | **N** | 47 | 47 | 37 | 47 | 46 | 47 | 46 | 47 | 47 | 47 |
| **Island** | **A** | 3 | 5 | 2 | 13 | 11 | 6 | 8 | 6 | 15 | 13 |
|  | ***H_O_*** | 0.468 | 0.170 | 0.162 | 0.745 | 0.674 | 0.340 | 0.543 | 0.426 | 0.511 | 0.532 |
|  | ***H_E_*** | 0.483 | 0.436 | 0.482 | 0.741 | 0.741 | 0.578 | 0.656 | 0.550 | 0.880 | 0.829 |
|  | ***F_IS_*** | 0.030 | *0.609* | *0.664* | -0.005 | 0.091 | *0.411* | 0.172 | 0.226 | *0.420* | *0.359* |
| **Man & Wife Rocks** | **N** | 28 | 26 | 25 | 28 | 28 | 28 | 25 | 25 | 28 | 27 |
|  | **A** | 8 | 5 | 5 | 11 | 13 | 6 | 11 | 7 | 12 | 10 |
|  | ***H_O_*** | 0.607 | 0.385 | 0.160 | 0.536 | 0.750 | 0.321 | 0.600 | 0.600 | 0.643 | 0.296 |
|  | ***H_E_*** | 0.770 | 0.524 | 0.446 | 0.798 | 0.864 | 0.651 | 0.809 | 0.698 | 0.855 | 0.809 |
|  | ***F_IS_*** | *0.211* | 0.267 | *0.641* | *0.329* | 0.131 | *0.506* | *0.258* | 0.141 | 0.248 | *0.634* |
| **Miall** | **N** | 45 | 45 | 37 | 45 | 45 | 45 | 43 | 45 | 45 | 45 |
| **Island** | **A** | 3 | 4 | 2 | 5 | 10 | 5 | 9 | 6 | 12 | 13 |
|  | ***H_O_*** | 0.600 | 0.333 | 0.189 | 0.533 | 0.756 | 0.356 | 0.512 | 0.711 | 0.378 | 0.644 |
|  | ***H_E_*** | 0.546 | 0.473 | 0.500 | 0.610 | 0.715 | 0.374 | 0.630 | 0.589 | 0.863 | 0.780 |
|  | ***F_IS_*** | -0.098 | *0.295* | *0.622* | *0.126* | -0.057 | 0.048 | 0.188 | -0.207 | *0.562* | 0.173 |
| **Nth Keppel** | **N** | 39 | 39 | 32 | 40 | 40 | 40 | 40 | 40 | 40 | 40 |
| **Island** | **A** | 3 | 3 | 2 | 6 | 10 | 7 | 7 | 5 | 12 | 11 |
|  | ***H_O_*** | 0.564 | 0.282 | 0.188 | 0.550 | 0.750 | 0.600 | 0.425 | 0.575 | 0.575 | 0.500 |
|  | ***H_E_*** | 0.558 | 0.344 | 0.451 | 0.533 | 0.718 | 0.609 | 0.664 | 0.637 | 0.799 | 0.762 |
|  | ***F_IS_*** | -0.011 | *0.181* | *0.584* | -0.033 | -0.044 | 0.014 | *0.360* | 0.098 | *0.280* | *0.344* |
| **Outer** | **N** | 49 | 50 | 46 | 50 | 50 | 50 | 47 | 48 | 49 | 50 |
| **Rocks** | **A** | 9 | 4 | 7 | 9 | 11 | 6 | 11 | 5 | 17 | 12 |
|  | ***H_O_*** | 0.429 | 0.340 | 0.174 | 0.620 | 0.780 | 0.340 | 0.447 | 0.500 | 0.592 | 0.440 |
|  | ***H_E_*** | 0.679 | 0.346 | 0.590 | 0.651 | 0.777 | 0.556 | 0.703 | 0.598 | 0.885 | 0.728 |
|  | ***F_IS_*** | *0.369* | 0.016 | *0.705* | 0.048 | -0.004 | *0.388* | *0.365* | 0.163 | *0.331* | *0.396* |
| **Passage** | **N** | 30 | 30 | 23 | 31 | 31 | 31 | 31 | 30 | 31 | 31 |
| **Rocks** | **A** | 5 | 6 | 3 | 13 | 11 | 4 | 8 | 4 | 12 | 7 |
|  | ***H_O_*** | 0.667 | 0.367 | 0.087 | 0.839 | 0.839 | 0.387 | 0.710 | 0.500 | 0.548 | 0.323 |
|  | ***H_E_*** | 0.561 | 0.503 | 0.529 | 0.849 | 0.772 | 0.546 | 0.691 | 0.603 | 0.856 | 0.662 |
|  | ***F_IS_*** | -0.189 | 0.272 | *0.836* | 0.012 | -0.087 | 0.291 | -0.026 | 0.171 | *0.360* | *0.513* |
