# Supplementary material for: A population genetic assessment of coral recovery on highly disturbed reefs of the Keppel Island archipelago in the southern Great Barrier Reef
Source: PeerJ. 2015 Jul 23;3:e1092. doi: 10.7717/peerj.1092 (PMC4517960; doi:10.7717/peerj.1092)
Supplement: Table S3 — Adjusted alpha was 0.011; significant values are highlighted in yellow. [file peerj-03-1092-s003.docx]

Table S3: Results of Linkage Disequilibrium tests for all loci in the nine Keppel Island populations for which new data were obtained in this study. Adjusted alpha was 0.011; significant values are highlighted in yellow.

| Population | Locus#1 | Locus#2 | P-Value | S.E. |
| --- | --- | --- | --- | --- |
| ---------- | ------- | ------- | -------- | -------- |
| Miall Island | Amil_002 | Amil_006 | 0.43085 | 0.015215 |
| Miall Island | Amil_002 | Amil_008 | 0.60645 | 0.00648 |
| Miall Island | Amil_006 | Amil_008 | 0.51286 | 0.008596 |
| Miall Island | Amil_002 | Amil_010 | 0.04873 | 0.007628 |
| Miall Island | Amil_006 | Amil_010 | 0.50271 | 0.024264 |
| Miall Island | Amil_008 | Amil_010 | 0.86524 | 0.005508 |
| Miall Island | Amil_002 | Amil_022 | 0.18864 | 0.023717 |
| Miall Island | Amil_006 | Amil_022 | 0.59638 | 0.031814 |
| Miall Island | Amil_008 | Amil_022 | 0.78578 | 0.010237 |
| Miall Island | Amil_010 | Amil_022 | 0.44571 | 0.034996 |
| Miall Island | Amil_002 | Amil_023 | 0.43259 | 0.016903 |
| Miall Island | Amil_006 | Amil_023 | 0.36658 | 0.023681 |
| Miall Island | Amil_008 | Amil_023 | 0.66886 | 0.007521 |
| Miall Island | Amil_010 | Amil_023 | 0.82692 | 0.017088 |
| Miall Island | Amil_022 | Amil_023 | 0.6091 | 0.031535 |
| Miall Island | Amil_002 | WGS_035 | 0.63428 | 0.027831 |
| Miall Island | Amil_006 | WGS_035 | 0.96921 | 0.011009 |
| Miall Island | Amil_008 | WGS_035 | 0.90425 | 0.006709 |
| Miall Island | Amil_010 | WGS_035 | 0.713 | 0.031297 |
| Miall Island | Amil_022 | WGS_035 | 0.41641 | 0.041831 |
| Miall Island | Amil_023 | WGS_035 | 0.05335 | 0.015193 |
| Miall Island | Amil_002 | WGS_134 | 0.42276 | 0.020212 |
| Miall Island | Amil_006 | WGS_134 | 0.69761 | 0.021898 |
| Miall Island | Amil_008 | WGS_134 | 0.06825 | 0.00501 |
| Miall Island | Amil_010 | WGS_134 | 0.37228 | 0.025685 |
| Miall Island | Amil_022 | WGS_134 | 0.43099 | 0.0345 |
| Miall Island | Amil_023 | WGS_134 | 0.72545 | 0.022609 |
| Miall Island | WGS_035 | WGS_134 | 0.44052 | 0.037974 |
| Miall Island | Amil_002 | WGS_189 | 0.27248 | 0.025847 |
| Miall Island | Amil_006 | WGS_189 | 0.516 | 0.035372 |
| Miall Island | Amil_008 | WGS_189 | 0.55791 | 0.015295 |
| Miall Island | Amil_010 | WGS_189 | 0.01823 | 0.008905 |
| Miall Island | Amil_022 | WGS_189 | 0.38082 | 0.042105 |
| Miall Island | Amil_023 | WGS_189 | 0.66662 | 0.031881 |
| Miall Island | WGS_035 | WGS_189 | 0.98581 | 0.010154 |
| Miall Island | WGS_134 | WGS_189 | 0.66768 | 0.032495 |
| Miall Island | Amil_002 | WGS_196 | 0.27656 | 0.028188 |
| Miall Island | Amil_006 | WGS_196 | 0.49001 | 0.036341 |
| Miall Island | Amil_008 | WGS_196 | 0.97572 | 0.00334 |
| Miall Island | Amil_010 | WGS_196 | 0.80306 | 0.026911 |
| Miall Island | Amil_022 | WGS_196 | 0.462 | 0.044183 |
| Miall Island | Amil_023 | WGS_196 | 0.50126 | 0.038889 |
| Miall Island | WGS_035 | WGS_196 | 0.56929 | 0.045466 |
| Miall Island | WGS_134 | WGS_196 | 0.01346 | 0.008628 |
| Miall Island | WGS_189 | WGS_196 | 0.80003 | 0.037757 |
| Barren Island | Amil_002 | Amil_006 | 0.28905 | 0.024503 |
| Barren Island | Amil_002 | Amil_008 | 0 | 0 |
| Barren Island | Amil_006 | Amil_008 | 0.56012 | 0.02073 |
| Barren Island | Amil_002 | Amil_010 | 0.13915 | 0.013065 |
| Barren Island | Amil_006 | Amil_010 | 0.0504 | 0.007657 |
| Barren Island | Amil_008 | Amil_010 | 0.33826 | 0.023538 |
| Barren Island | Amil_002 | Amil_022 | 0 | 0 |
| Barren Island | Amil_006 | Amil_022 | 0.30185 | 0.023932 |
| Barren Island | Amil_008 | Amil_022 | 0.03152 | 0.012664 |
| Barren Island | Amil_010 | Amil_022 | 0.26959 | 0.02133 |
| Barren Island | Amil_002 | Amil_023 | 0.01126 | 0.004768 |
| Barren Island | Amil_006 | Amil_023 | 0.36606 | 0.022342 |
| Barren Island | Amil_008 | Amil_023 | 0.4807 | 0.031782 |
| Barren Island | Amil_010 | Amil_023 | 0 | 0 |
| Barren Island | Amil_022 | Amil_023 | 0.00168 | 0.001563 |
| Barren Island | Amil_002 | WGS_035 | 0.62511 | 0.032761 |
| Barren Island | Amil_006 | WGS_035 | 0.25355 | 0.029204 |
| Barren Island | Amil_008 | WGS_035 | 0.97395 | 0.00501 |
| Barren Island | Amil_010 | WGS_035 | 0.14573 | 0.019571 |
| Barren Island | Amil_022 | WGS_035 | 0.0051 | 0.0051 |
| Barren Island | Amil_023 | WGS_035 | 0.48723 | 0.03041 |
| Barren Island | Amil_002 | WGS_134 | 0.67598 | 0.026651 |
| Barren Island | Amil_006 | WGS_134 | 0.77257 | 0.017853 |
| Barren Island | Amil_008 | WGS_134 | 0.94161 | 0.010059 |
| Barren Island | Amil_010 | WGS_134 | 0.35393 | 0.016998 |
| Barren Island | Amil_022 | WGS_134 | 0.30422 | 0.027847 |
| Barren Island | Amil_023 | WGS_134 | 0.31627 | 0.019084 |
| Barren Island | WGS_035 | WGS_134 | 0.83797 | 0.01994 |
| Barren Island | Amil_002 | WGS_189 | 0.05723 | 0.017912 |
| Barren Island | Amil_006 | WGS_189 | 0.88657 | 0.019349 |
| Barren Island | Amil_008 | WGS_189 | 0.1682 | 0.030192 |
| Barren Island | Amil_010 | WGS_189 | 0.26624 | 0.023606 |
| Barren Island | Amil_022 | WGS_189 | 0.01715 | 0.01085 |
| Barren Island | Amil_023 | WGS_189 | 0.04277 | 0.013638 |
| Barren Island | WGS_035 | WGS_189 | 0.27673 | 0.032437 |
| Barren Island | WGS_134 | WGS_189 | 0.01885 | 0.007009 |
| Barren Island | Amil_002 | WGS_196 | 0.60982 | 0.030555 |
| Barren Island | Amil_006 | WGS_196 | 0.07837 | 0.013686 |
| Barren Island | Amil_008 | WGS_196 | 0.03422 | 0.008234 |
| Barren Island | Amil_010 | WGS_196 | 0.07667 | 0.0129 |
| Barren Island | Amil_022 | WGS_196 | 0.9931 | 0.003418 |
| Barren Island | Amil_023 | WGS_196 | 0.12971 | 0.017514 |
| Barren Island | WGS_035 | WGS_196 | 0.0239 | 0.01184 |
| Barren Island | WGS_134 | WGS_196 | 0.81428 | 0.021064 |
| Barren Island | WGS_189 | WGS_196 | 0.69571 | 0.03318 |
| Halftide Rocks | Amil_002 | Amil_006 | 0.06842 | 0.013676 |
| Halftide Rocks | Amil_002 | Amil_008 | 0.68163 | 0.01043 |
| Halftide Rocks | Amil_006 | Amil_008 | 0.50299 | 0.019165 |
| Halftide Rocks | Amil_002 | Amil_010 | 0.08459 | 0.0142 |
| Halftide Rocks | Amil_006 | Amil_010 | 0.8481 | 0.024949 |
| Halftide Rocks | Amil_008 | Amil_010 | 0.06071 | 0.01045 |
| Halftide Rocks | Amil_002 | Amil_022 | 0.1226 | 0.018367 |
| Halftide Rocks | Amil_006 | Amil_022 | 0.09636 | 0.019615 |
| Halftide Rocks | Amil_008 | Amil_022 | 0.02366 | 0.005608 |
| Halftide Rocks | Amil_010 | Amil_022 | 0.14283 | 0.025554 |
| Halftide Rocks | Amil_002 | Amil_023 | 0.17859 | 0.014793 |
| Halftide Rocks | Amil_006 | Amil_023 | 0.73268 | 0.021348 |
| Halftide Rocks | Amil_008 | Amil_023 | 0.28822 | 0.009932 |
| Halftide Rocks | Amil_010 | Amil_023 | 0.45715 | 0.028414 |
| Halftide Rocks | Amil_022 | Amil_023 | 0.72467 | 0.023293 |
| Halftide Rocks | Amil_002 | WGS_035 | 0.80219 | 0.029054 |
| Halftide Rocks | Amil_006 | WGS_035 | 0.33863 | 0.040598 |
| Halftide Rocks | Amil_008 | WGS_035 | 0.0764 | 0.016332 |
| Halftide Rocks | Amil_010 | WGS_035 | 0.59142 | 0.041801 |
| Halftide Rocks | Amil_022 | WGS_035 | 0.15797 | 0.031004 |
| Halftide Rocks | Amil_023 | WGS_035 | 0.26679 | 0.032645 |
| Halftide Rocks | Amil_002 | WGS_134 | 0.05388 | 0.010233 |
| Halftide Rocks | Amil_006 | WGS_134 | 0.25938 | 0.027947 |
| Halftide Rocks | Amil_008 | WGS_134 | 0.71413 | 0.016105 |
| Halftide Rocks | Amil_010 | WGS_134 | 0.47824 | 0.037723 |
| Halftide Rocks | Amil_022 | WGS_134 | 0.12959 | 0.022837 |
| Halftide Rocks | Amil_023 | WGS_134 | 0 | 0 |
| Halftide Rocks | WGS_035 | WGS_134 | 0.01781 | 0.008062 |
| Halftide Rocks | Amil_002 | WGS_189 | 0.07965 | 0.018655 |
| Halftide Rocks | Amil_006 | WGS_189 | 0.64522 | 0.038067 |
| Halftide Rocks | Amil_008 | WGS_189 | 0.2929 | 0.02552 |
| Halftide Rocks | Amil_010 | WGS_189 | 0.00684 | 0.004146 |
| Halftide Rocks | Amil_022 | WGS_189 | 0.03299 | 0.012963 |
| Halftide Rocks | Amil_023 | WGS_189 | 0.06524 | 0.014216 |
| Halftide Rocks | WGS_035 | WGS_189 | 0 | 0 |
| Halftide Rocks | WGS_134 | WGS_189 | 0.00008 | 0.00008 |
| Halftide Rocks | Amil_002 | WGS_196 | 0.27627 | 0.029609 |
| Halftide Rocks | Amil_006 | WGS_196 | 0.41899 | 0.040022 |
| Halftide Rocks | Amil_008 | WGS_196 | 0.13215 | 0.016959 |
| Halftide Rocks | Amil_010 | WGS_196 | 0.05197 | 0.017476 |
| Halftide Rocks | Amil_022 | WGS_196 | 0.06608 | 0.017408 |
| Halftide Rocks | Amil_023 | WGS_196 | 0.229 | 0.031357 |
| Halftide Rocks | WGS_035 | WGS_196 | 0.29403 | 0.04273 |
| Halftide Rocks | WGS_134 | WGS_196 | 0.0097 | 0.005739 |
| Halftide Rocks | WGS_189 | WGS_196 | 0 | 0 |
| Halfway Island | Amil_002 | Amil_006 | 0.24855 | 0.022715 |
| Halfway Island | Amil_002 | Amil_008 | 0.63294 | 0.019069 |
| Halfway Island | Amil_006 | Amil_008 | 0.06603 | 0.012272 |
| Halfway Island | Amil_002 | Amil_010 | 0.66969 | 0.028301 |
| Halfway Island | Amil_006 | Amil_010 | 0.81606 | 0.027316 |
| Halfway Island | Amil_008 | Amil_010 | 0.92313 | 0.014398 |
| Halfway Island | Amil_002 | Amil_022 | 0.42804 | 0.030032 |
| Halfway Island | Amil_006 | Amil_022 | 0.27268 | 0.031862 |
| Halfway Island | Amil_008 | Amil_022 | 0.51608 | 0.030373 |
| Halfway Island | Amil_010 | Amil_022 | 0.48359 | 0.041502 |
| Halfway Island | Amil_002 | Amil_023 | 0.62915 | 0.021927 |
| Halfway Island | Amil_006 | Amil_023 | 0.59032 | 0.028699 |
| Halfway Island | Amil_008 | Amil_023 | 0.08595 | 0.012222 |
| Halfway Island | Amil_010 | Amil_023 | 0.31553 | 0.032043 |
| Halfway Island | Amil_022 | Amil_023 | 0.08954 | 0.016494 |
| Halfway Island | Amil_002 | WGS_035 | 0.72835 | 0.031927 |
| Halfway Island | Amil_006 | WGS_035 | 0.0257 | 0.01129 |
| Halfway Island | Amil_008 | WGS_035 | 0.30632 | 0.028913 |
| Halfway Island | Amil_010 | WGS_035 | 0.7903 | 0.032547 |
| Halfway Island | Amil_022 | WGS_035 | 0.60802 | 0.043501 |
| Halfway Island | Amil_023 | WGS_035 | 0.02906 | 0.010905 |
| Halfway Island | Amil_002 | WGS_134 | 0.95272 | 0.006976 |
| Halfway Island | Amil_006 | WGS_134 | 0.75453 | 0.022663 |
| Halfway Island | Amil_008 | WGS_134 | 0.67845 | 0.016534 |
| Halfway Island | Amil_010 | WGS_134 | 0.70426 | 0.029052 |
| Halfway Island | Amil_022 | WGS_134 | 0.05241 | 0.011976 |
| Halfway Island | Amil_023 | WGS_134 | 0.8995 | 0.012266 |
| Halfway Island | WGS_035 | WGS_134 | 0.77616 | 0.027903 |
| Halfway Island | Amil_002 | WGS_189 | 0.53905 | 0.036169 |
| Halfway Island | Amil_006 | WGS_189 | 0.3707 | 0.038227 |
| Halfway Island | Amil_008 | WGS_189 | 0.12396 | 0.022548 |
| Halfway Island | Amil_010 | WGS_189 | 0.54264 | 0.042189 |
| Halfway Island | Amil_022 | WGS_189 | 0.95406 | 0.014956 |
| Halfway Island | Amil_023 | WGS_189 | 0.23814 | 0.032057 |
| Halfway Island | WGS_035 | WGS_189 | 0.01643 | 0.007258 |
| Halfway Island | WGS_134 | WGS_189 | 0.28736 | 0.032297 |
| Halfway Island | Amil_002 | WGS_196 | 0.3686 | 0.034508 |
| Halfway Island | Amil_006 | WGS_196 | 0.09324 | 0.025798 |
| Halfway Island | Amil_008 | WGS_196 | 0.20145 | 0.02787 |
| Halfway Island | Amil_010 | WGS_196 | 0.67032 | 0.039699 |
| Halfway Island | Amil_022 | WGS_196 | 0.2604 | 0.038966 |
| Halfway Island | Amil_023 | WGS_196 | 0.78453 | 0.033581 |
| Halfway Island | WGS_035 | WGS_196 | 0.25091 | 0.040624 |
| Halfway Island | WGS_134 | WGS_196 | 0.78511 | 0.03035 |
| Halfway Island | WGS_189 | WGS_196 | 0.25996 | 0.041351 |
| Outer Rocks | Amil_002 | Amil_006 | 0.97515 | 0.007984 |
| Outer Rocks | Amil_002 | Amil_008 | 0.00997 | 0.007638 |
| Outer Rocks | Amil_006 | Amil_008 | 0.8302 | 0.016713 |
| Outer Rocks | Amil_002 | Amil_010 | 0.11299 | 0.022943 |
| Outer Rocks | Amil_006 | Amil_010 | 0.15806 | 0.019577 |
| Outer Rocks | Amil_008 | Amil_010 | 0.08753 | 0.01517 |
| Outer Rocks | Amil_002 | Amil_022 | 0.55292 | 0.043198 |
| Outer Rocks | Amil_006 | Amil_022 | 0.78982 | 0.026846 |
| Outer Rocks | Amil_008 | Amil_022 | 0.10203 | 0.021076 |
| Outer Rocks | Amil_010 | Amil_022 | 0.06903 | 0.016957 |
| Outer Rocks | Amil_002 | Amil_023 | 0 | 0 |
| Outer Rocks | Amil_006 | Amil_023 | 0.93243 | 0.00961 |
| Outer Rocks | Amil_008 | Amil_023 | 0.0432 | 0.009775 |
| Outer Rocks | Amil_010 | Amil_023 | 0.00182 | 0.00182 |
| Outer Rocks | Amil_022 | Amil_023 | 0.29445 | 0.034589 |
| Outer Rocks | Amil_002 | WGS_035 | 0.30547 | 0.041605 |
| Outer Rocks | Amil_006 | WGS_035 | 0.96828 | 0.009897 |
| Outer Rocks | Amil_008 | WGS_035 | 0.20571 | 0.030858 |
| Outer Rocks | Amil_010 | WGS_035 | 0.26452 | 0.035881 |
| Outer Rocks | Amil_022 | WGS_035 | 0.3215 | 0.043244 |
| Outer Rocks | Amil_023 | WGS_035 | 0.07295 | 0.020392 |
| Outer Rocks | Amil_002 | WGS_134 | 0.71404 | 0.021729 |
| Outer Rocks | Amil_006 | WGS_134 | 0.51244 | 0.019186 |
| Outer Rocks | Amil_008 | WGS_134 | 0.87312 | 0.011263 |
| Outer Rocks | Amil_010 | WGS_134 | 0.48177 | 0.025528 |
| Outer Rocks | Amil_022 | WGS_134 | 0.5203 | 0.033701 |
| Outer Rocks | Amil_023 | WGS_134 | 0.83056 | 0.012162 |
| Outer Rocks | WGS_035 | WGS_134 | 0.54955 | 0.031529 |
| Outer Rocks | Amil_002 | WGS_189 | 0.93385 | 0.020706 |
| Outer Rocks | Amil_006 | WGS_189 | 0.48699 | 0.036206 |
| Outer Rocks | Amil_008 | WGS_189 | 0.0894 | 0.021117 |
| Outer Rocks | Amil_010 | WGS_189 | 0.25574 | 0.03792 |
| Outer Rocks | Amil_022 | WGS_189 | 0.7683 | 0.040647 |
| Outer Rocks | Amil_023 | WGS_189 | 0.17485 | 0.026875 |
| Outer Rocks | WGS_035 | WGS_189 | 0.36782 | 0.045561 |
| Outer Rocks | WGS_134 | WGS_189 | 0.50217 | 0.033453 |
| Outer Rocks | Amil_002 | WGS_196 | 0.18372 | 0.034593 |
| Outer Rocks | Amil_006 | WGS_196 | 0.06045 | 0.019089 |
| Outer Rocks | Amil_008 | WGS_196 | 0.34954 | 0.036161 |
| Outer Rocks | Amil_010 | WGS_196 | 0.20444 | 0.034073 |
| Outer Rocks | Amil_022 | WGS_196 | 0.93196 | 0.021927 |
| Outer Rocks | Amil_023 | WGS_196 | 0.46658 | 0.037228 |
| Outer Rocks | WGS_035 | WGS_196 | 0.31992 | 0.041863 |
| Outer Rocks | WGS_134 | WGS_196 | 0.59778 | 0.032061 |
| Outer Rocks | WGS_189 | WGS_196 | 0.5885 | 0.046552 |
| Man & Wife Rocks | Amil_002 | Amil_006 | 0.53144 | 0.027389 |
| Man & Wife Rocks | Amil_002 | Amil_008 | 0.07123 | 0.01636 |
| Man & Wife Rocks | Amil_006 | Amil_008 | 0.60074 | 0.020665 |
| Man & Wife Rocks | Amil_002 | Amil_010 | 0.34454 | 0.036796 |
| Man & Wife Rocks | Amil_006 | Amil_010 | 0.29157 | 0.028037 |
| Man & Wife Rocks | Amil_008 | Amil_010 | 0.19098 | 0.021361 |
| Man & Wife Rocks | Amil_002 | Amil_022 | 0.06473 | 0.01826 |
| Man & Wife Rocks | Amil_006 | Amil_022 | 0.20021 | 0.020961 |
| Man & Wife Rocks | Amil_008 | Amil_022 | 0.57593 | 0.031258 |
| Man & Wife Rocks | Amil_010 | Amil_022 | 0.01608 | 0.008193 |
| Man & Wife Rocks | Amil_002 | Amil_023 | 0.00119 | 0.00119 |
| Man & Wife Rocks | Amil_006 | Amil_023 | 0.09919 | 0.011313 |
| Man & Wife Rocks | Amil_008 | Amil_023 | 0.02066 | 0.004564 |
| Man & Wife Rocks | Amil_010 | Amil_023 | 0 | 0 |
| Man & Wife Rocks | Amil_022 | Amil_023 | 0.37964 | 0.032083 |
| Man & Wife Rocks | Amil_002 | WGS_035 | 0.44107 | 0.038184 |
| Man & Wife Rocks | Amil_006 | WGS_035 | 0.72246 | 0.02723 |
| Man & Wife Rocks | Amil_008 | WGS_035 | 0.09972 | 0.01396 |
| Man & Wife Rocks | Amil_010 | WGS_035 | 0.55266 | 0.043618 |
| Man & Wife Rocks | Amil_022 | WGS_035 | 1 | 0 |
| Man & Wife Rocks | Amil_023 | WGS_035 | 0.1646 | 0.030687 |
| Man & Wife Rocks | Amil_002 | WGS_134 | 0.31789 | 0.02601 |
| Man & Wife Rocks | Amil_006 | WGS_134 | 0.00956 | 0.004686 |
| Man & Wife Rocks | Amil_008 | WGS_134 | 0.31562 | 0.017852 |
| Man & Wife Rocks | Amil_010 | WGS_134 | 0.57457 | 0.033015 |
| Man & Wife Rocks | Amil_022 | WGS_134 | 0.33774 | 0.033877 |
| Man & Wife Rocks | Amil_023 | WGS_134 | 0.10315 | 0.016473 |
| Man & Wife Rocks | WGS_035 | WGS_134 | 1 | 0 |
| Man & Wife Rocks | Amil_002 | WGS_189 | 0.34059 | 0.039777 |
| Man & Wife Rocks | Amil_006 | WGS_189 | 0.84835 | 0.022829 |
| Man & Wife Rocks | Amil_008 | WGS_189 | 0.77225 | 0.028778 |
| Man & Wife Rocks | Amil_010 | WGS_189 | 0.56269 | 0.045556 |
| Man & Wife Rocks | Amil_022 | WGS_189 | 0.00455 | 0.003972 |
| Man & Wife Rocks | Amil_023 | WGS_189 | 0.88822 | 0.021687 |
| Man & Wife Rocks | WGS_035 | WGS_189 | 1 | 0 |
| Man & Wife Rocks | WGS_134 | WGS_189 | 0.51834 | 0.038551 |
| Man & Wife Rocks | Amil_002 | WGS_196 | 0.51822 | 0.038071 |
| Man & Wife Rocks | Amil_006 | WGS_196 | 0.31592 | 0.026276 |
| Man & Wife Rocks | Amil_008 | WGS_196 | 0.30571 | 0.027899 |
| Man & Wife Rocks | Amil_010 | WGS_196 | 0.55996 | 0.042308 |
| Man & Wife Rocks | Amil_022 | WGS_196 | 0.4524 | 0.044579 |
| Man & Wife Rocks | Amil_023 | WGS_196 | 0.23845 | 0.030567 |
| Man & Wife Rocks | WGS_035 | WGS_196 | 0.62897 | 0.040073 |
| Man & Wife Rocks | WGS_134 | WGS_196 | 0.16494 | 0.023977 |
| Man & Wife Rocks | WGS_189 | WGS_196 | 1 | 0 |
| North Keppel Island | Amil_002 | Amil_006 | 0.70682 | 0.010562 |
| North Keppel Island | Amil_002 | Amil_008 | 0.29658 | 0.007455 |
| North Keppel Island | Amil_006 | Amil_008 | 0.65956 | 0.006377 |
| North Keppel Island | Amil_002 | Amil_010 | 0.92812 | 0.009977 |
| North Keppel Island | Amil_006 | Amil_010 | 0.81477 | 0.012812 |
| North Keppel Island | Amil_008 | Amil_010 | 0.24708 | 0.008612 |
| North Keppel Island | Amil_002 | Amil_022 | 0.4779 | 0.030778 |
| North Keppel Island | Amil_006 | Amil_022 | 0.04955 | 0.009587 |
| North Keppel Island | Amil_008 | Amil_022 | 0.16857 | 0.009804 |
| North Keppel Island | Amil_010 | Amil_022 | 0.28846 | 0.032755 |
| North Keppel Island | Amil_002 | Amil_023 | 0.07953 | 0.011075 |
| North Keppel Island | Amil_006 | Amil_023 | 0.47507 | 0.019437 |
| North Keppel Island | Amil_008 | Amil_023 | 0.70742 | 0.010312 |
| North Keppel Island | Amil_010 | Amil_023 | 0.17547 | 0.021129 |
| North Keppel Island | Amil_022 | Amil_023 | 0.73115 | 0.028922 |
| North Keppel Island | Amil_002 | WGS_035 | 0.36629 | 0.029721 |
| North Keppel Island | Amil_006 | WGS_035 | 0.26568 | 0.023555 |
| North Keppel Island | Amil_008 | WGS_035 | 0.53134 | 0.015728 |
| North Keppel Island | Amil_010 | WGS_035 | 0.21233 | 0.027599 |
| North Keppel Island | Amil_022 | WGS_035 | 0.13815 | 0.027532 |
| North Keppel Island | Amil_023 | WGS_035 | 0.89478 | 0.02207 |
| North Keppel Island | Amil_002 | WGS_134 | 0.72853 | 0.016555 |
| North Keppel Island | Amil_006 | WGS_134 | 0.30818 | 0.012583 |
| North Keppel Island | Amil_008 | WGS_134 | 0.86015 | 0.005116 |
| North Keppel Island | Amil_010 | WGS_134 | 0.11115 | 0.015861 |
| North Keppel Island | Amil_022 | WGS_134 | 0.18152 | 0.023659 |
| North Keppel Island | Amil_023 | WGS_134 | 0.16658 | 0.021506 |
| North Keppel Island | WGS_035 | WGS_134 | 0.6401 | 0.030277 |
| North Keppel Island | Amil_002 | WGS_189 | 0.40686 | 0.036485 |
| North Keppel Island | Amil_006 | WGS_189 | 0.5423 | 0.028739 |
| North Keppel Island | Amil_008 | WGS_189 | 0.31162 | 0.015714 |
| North Keppel Island | Amil_010 | WGS_189 | 0.00443 | 0.003129 |
| North Keppel Island | Amil_022 | WGS_189 | 0.38803 | 0.04548 |
| North Keppel Island | Amil_023 | WGS_189 | 0.30166 | 0.037086 |
| North Keppel Island | WGS_035 | WGS_189 | 0.48629 | 0.04638 |
| North Keppel Island | WGS_134 | WGS_189 | 0.42498 | 0.03703 |
| North Keppel Island | Amil_002 | WGS_196 | 0.05897 | 0.011904 |
| North Keppel Island | Amil_006 | WGS_196 | 0.91474 | 0.010642 |
| North Keppel Island | Amil_008 | WGS_196 | 0.75145 | 0.012939 |
| North Keppel Island | Amil_010 | WGS_196 | 0.67214 | 0.033895 |
| North Keppel Island | Amil_022 | WGS_196 | 0.79629 | 0.029006 |
| North Keppel Island | Amil_023 | WGS_196 | 0.62772 | 0.034836 |
| North Keppel Island | WGS_035 | WGS_196 | 0.18508 | 0.030128 |
| North Keppel Island | WGS_134 | WGS_196 | 0.00648 | 0.003508 |
| North Keppel Island | WGS_189 | WGS_196 | 0.73086 | 0.039647 |
| Humpy Island | Amil_002 | Amil_006 | 0.80044 | 0.014974 |
| Humpy Island | Amil_002 | Amil_008 | 0.74638 | 0.003754 |
| Humpy Island | Amil_006 | Amil_008 | 0.02752 | 0.002645 |
| Humpy Island | Amil_002 | Amil_010 | 0.84745 | 0.019424 |
| Humpy Island | Amil_006 | Amil_010 | 0.46834 | 0.034355 |
| Humpy Island | Amil_008 | Amil_010 | 0.22527 | 0.013323 |
| Humpy Island | Amil_002 | Amil_022 | 0.26956 | 0.025114 |
| Humpy Island | Amil_006 | Amil_022 | 0.00272 | 0.002436 |
| Humpy Island | Amil_008 | Amil_022 | 0.84337 | 0.008298 |
| Humpy Island | Amil_010 | Amil_022 | 0.0914 | 0.026639 |
| Humpy Island | Amil_002 | Amil_023 | 0.62019 | 0.020422 |
| Humpy Island | Amil_006 | Amil_023 | 0.2284 | 0.026213 |
| Humpy Island | Amil_008 | Amil_023 | 0.97157 | 0.002233 |
| Humpy Island | Amil_010 | Amil_023 | 0.37581 | 0.038073 |
| Humpy Island | Amil_022 | Amil_023 | 0.75812 | 0.036731 |
| Humpy Island | Amil_002 | WGS_035 | 0.43725 | 0.023197 |
| Humpy Island | Amil_006 | WGS_035 | 0.19137 | 0.030989 |
| Humpy Island | Amil_008 | WGS_035 | 0.8709 | 0.007764 |
| Humpy Island | Amil_010 | WGS_035 | 0.9717 | 0.01509 |
| Humpy Island | Amil_022 | WGS_035 | 0.99823 | 0.000989 |
| Humpy Island | Amil_023 | WGS_035 | 0.90187 | 0.019811 |
| Humpy Island | Amil_002 | WGS_134 | 0.09749 | 0.015097 |
| Humpy Island | Amil_006 | WGS_134 | 0.12513 | 0.021618 |
| Humpy Island | Amil_008 | WGS_134 | 0.78214 | 0.009316 |
| Humpy Island | Amil_010 | WGS_134 | 0.15591 | 0.031674 |
| Humpy Island | Amil_022 | WGS_134 | 0.57761 | 0.042021 |
| Humpy Island | Amil_023 | WGS_134 | 0.29058 | 0.029823 |
| Humpy Island | WGS_035 | WGS_134 | 0.8437 | 0.028526 |
| Humpy Island | Amil_002 | WGS_189 | 0.11763 | 0.019173 |
| Humpy Island | Amil_006 | WGS_189 | 0.88279 | 0.024692 |
| Humpy Island | Amil_008 | WGS_189 | 0.85337 | 0.009341 |
| Humpy Island | Amil_010 | WGS_189 | 0.15988 | 0.035582 |
| Humpy Island | Amil_022 | WGS_189 | 0.52411 | 0.046862 |
| Humpy Island | Amil_023 | WGS_189 | 0.70062 | 0.03957 |
| Humpy Island | WGS_035 | WGS_189 | 0.15294 | 0.033276 |
| Humpy Island | WGS_134 | WGS_189 | 0.3608 | 0.041062 |
| Humpy Island | Amil_002 | WGS_196 | 0.00742 | 0.003588 |
| Humpy Island | Amil_006 | WGS_196 | 0.64203 | 0.038314 |
| Humpy Island | Amil_008 | WGS_196 | 0.58157 | 0.019252 |
| Humpy Island | Amil_010 | WGS_196 | 0.05471 | 0.021516 |
| Humpy Island | Amil_022 | WGS_196 | 0.85548 | 0.031458 |
| Humpy Island | Amil_023 | WGS_196 | 0.0208 | 0.012356 |
| Humpy Island | WGS_035 | WGS_196 | 0.1223 | 0.029045 |
| Humpy Island | WGS_134 | WGS_196 | 0.02996 | 0.014021 |
| Humpy Island | WGS_189 | WGS_196 | 0.32068 | 0.046153 |
| Passage Rocks | Amil_002 | Amil_006 | 0.56651 | 0.023517 |
| Passage Rocks | Amil_002 | Amil_008 | 0.00003 | 0.00003 |
| Passage Rocks | Amil_006 | Amil_008 | 0.53457 | 0.015368 |
| Passage Rocks | Amil_002 | Amil_010 | 0.99521 | 0.002052 |
| Passage Rocks | Amil_006 | Amil_010 | 0.55872 | 0.032744 |
| Passage Rocks | Amil_008 | Amil_010 | 0.98971 | 0.002585 |
| Passage Rocks | Amil_002 | Amil_022 | 0.39496 | 0.031006 |
| Passage Rocks | Amil_006 | Amil_022 | 0.57876 | 0.034728 |
| Passage Rocks | Amil_008 | Amil_022 | 0.96745 | 0.006298 |
| Passage Rocks | Amil_010 | Amil_022 | 0.21754 | 0.03568 |
| Passage Rocks | Amil_002 | Amil_023 | 0.01285 | 0.002703 |
| Passage Rocks | Amil_006 | Amil_023 | 0.98816 | 0.001899 |
| Passage Rocks | Amil_008 | Amil_023 | 0.15203 | 0.005304 |
| Passage Rocks | Amil_010 | Amil_023 | 0.42243 | 0.021179 |
| Passage Rocks | Amil_022 | Amil_023 | 0.07296 | 0.010543 |
| Passage Rocks | Amil_002 | WGS_035 | 0.89895 | 0.016686 |
| Passage Rocks | Amil_006 | WGS_035 | 0.64071 | 0.033635 |
| Passage Rocks | Amil_008 | WGS_035 | 0.1179 | 0.014446 |
| Passage Rocks | Amil_010 | WGS_035 | 0.20138 | 0.037772 |
| Passage Rocks | Amil_022 | WGS_035 | 0.20989 | 0.035698 |
| Passage Rocks | Amil_023 | WGS_035 | 0.65585 | 0.022664 |
| Passage Rocks | Amil_002 | WGS_134 | 0.8329 | 0.012226 |
| Passage Rocks | Amil_006 | WGS_134 | 0.26177 | 0.020586 |
| Passage Rocks | Amil_008 | WGS_134 | 0.01685 | 0.002042 |
| Passage Rocks | Amil_010 | WGS_134 | 0.75009 | 0.024064 |
| Passage Rocks | Amil_022 | WGS_134 | 0.3533 | 0.028173 |
| Passage Rocks | Amil_023 | WGS_134 | 0.97989 | 0.002263 |
| Passage Rocks | WGS_035 | WGS_134 | 0.27987 | 0.028638 |
| Passage Rocks | Amil_002 | WGS_189 | 0.74563 | 0.023611 |
| Passage Rocks | Amil_006 | WGS_189 | 0.52599 | 0.03548 |
| Passage Rocks | Amil_008 | WGS_189 | 0.98433 | 0.003606 |
| Passage Rocks | Amil_010 | WGS_189 | 1 | 0 |
| Passage Rocks | Amil_022 | WGS_189 | 1 | 0 |
| Passage Rocks | Amil_023 | WGS_189 | 0.91785 | 0.010649 |
| Passage Rocks | WGS_035 | WGS_189 | 1 | 0 |
| Passage Rocks | WGS_134 | WGS_189 | 0.36987 | 0.029457 |
| Passage Rocks | Amil_002 | WGS_196 | 0.94504 | 0.01135 |
| Passage Rocks | Amil_006 | WGS_196 | 0.74845 | 0.029498 |
| Passage Rocks | Amil_008 | WGS_196 | 0.23209 | 0.013618 |
| Passage Rocks | Amil_010 | WGS_196 | 0.66754 | 0.038538 |
| Passage Rocks | Amil_022 | WGS_196 | 0.4064 | 0.042237 |
| Passage Rocks | Amil_023 | WGS_196 | 0.86362 | 0.015906 |
| Passage Rocks | WGS_035 | WGS_196 | 0 | 0 |
| Passage Rocks | WGS_134 | WGS_196 | 0.01789 | 0.012027 |
| Passage Rocks | WGS_189 | WGS_196 | 0.74448 | 0.03767 |
