# Supplementary material for: A population genetic assessment of coral recovery on highly disturbed reefs of the Keppel Island archipelago in the southern Great Barrier Reef
Source: PeerJ. 2015 Jul 23;3:e1092. doi: 10.7717/peerj.1092 (PMC4517960; doi:10.7717/peerj.1092)
Supplement: Table S4 — Most values are statistically significant; non-significant values have shaded background, and p-values larger than adjusted α are printed in bold face (adjusted α = 0.012). [file peerj-03-1092-s004.docx]

Table S4: Pairwise *F*_ST_ values below diagonal, p-values above diagonal. Most values are statistically significant; non-significant values have shaded background, and p-values larger than adjusted α are printed in bold face (adjusted α = 0.012).

|  | **Barren Island** | **Halftide Rocks** | **Halfway Island** | **Humpy Island** | **Man & Wife Rocks** | **Miall Island** | **Nth Keppel Island** | **Outer Rocks** | **Passage Rocks** |
| --- | --- | --- | --- | --- | --- | --- | --- | --- | --- |
| **Barren** |  | <0.001 | <0.001 | <0.001 | <0.001 | <0.001 | <0.001 | <0.001 | <0.001 |
| **Halftide** | 0.189 |  | <0.001 | <0.001 | <0.001 | <0.001 | <0.001 | <0.001 | <0.001 |
| **Halfway** | 0.158 | 0.010 |  | 0.002 | <0.001 | **0.033** | **0.161** | 0.004 | <0.001 |
| **Humpy** | 0.126 | 0.035 | 0.018 |  | <0.001 | <0.001 | 0.005 | <0.001 | <0.001 |
| **Man&Wife** | 0.094 | 0.061 | 0.044 | 0.042 |  | 0.001 | <0.001 | <0.001 | <0.001 |
| **Miall** | 0.168 | 0.037 | 0.017 | 0.008 | 0.060 |  | **0.030** | <0.001 | <0.001 |
| **Nth Keppel** | 0.155 | 0.032 | 0.005 | 0.015 | 0.049 | 0.007 |  | 0.003 | <0.001 |
| **Outer** | 0.115 | 0.032 | 0.009 | 0.017 | 0.026 | 0.012 | 0.009 |  | <0.001 |
| **Passage** | 0.151 | 0.105 | 0.082 | 0.024 | 0.075 | 0.080 | 0.071 | <0.001 |  |
