# Supplementary material for: A population genetic assessment of coral recovery on highly disturbed reefs of the Keppel Island archipelago in the southern Great Barrier Reef
Source: PeerJ. 2015 Jul 23;3:e1092. doi: 10.7717/peerj.1092 (PMC4517960; doi:10.7717/peerj.1092)
Supplement: Table S5 — Assignment probabilities >0.1 are highlighted. [file peerj-03-1092-s005.docx]

Table S5: Assignment probabilities of putative recent migrants as identified in GeneClass2.

Assignment probabilities >0.1 are highlighted.

|  | **Barren** | **Halftide** | **Halfway** | **Humpy** | **Man & Wife** | **Miall** | **Nth Keppel** | **Outer** | **Passage** |
| --- | --- | --- | --- | --- | --- | --- | --- | --- | --- |
| **Assigned sample** | **probability** | **probability** | **probability** | **probability** | **probability** | **probability** | **probability** | **probability** | **probability** |
| **Barren** | 0.0262 | 0 | 0.0017 | 0.0027 | 0.3863 | 0.0002 | 0.0009 | 0.1773 | 0.0036 |
| **Barren** | 0.0775 | 0.9161 | 0.8173 | 0.8513 | 0.9892 | 0.639 | 0.7479 | 0.802 | 0.3887 |
| **Barren** | 0.0058 | 0.0208 | 0.0783 | 0.023 | 0.4072 | 0.0245 | 0.0096 | 0.0249 | 0.0209 |
| **Barren** | 0.0134 | 0.1092 | 0.0924 | 0.0855 | 0.5692 | 0.1431 | 0.0205 | 0.6381 | 0.0676 |
| **Barren** | 0.005 | 0.2918 | 0.4441 | 0.6364 | 0.5676 | 0.1219 | 0.1257 | 0.5095 | 0.3896 |
| **Man & Wife** | 0.0065 | 0.0097 | 0.0347 | 0.0323 | 0.1829 | 0.022 | 0.0468 | 0.2165 | 0.0558 |
| **Man & Wife** | 0.0001 | 0.0002 | 0.0084 | 0.0032 | 0.0308 | 0.0049 | 0.0061 | 0.0147 | 0.0373 |
| **Man & Wife** | 0.0081 | 0 | 0 | 0 | 0.0092 | 0 | 0 | 0.0059 | 0 |
| **Outer** | 0.3393 | 0 | 0 | 0 | 0.0014 | 0 | 0 | 0.0014 | 0 |
| **Outer** | 0.2208 | 0 | 0 | 0 | 0.007 | 0 | 0 | 0.0025 | 0 |
| **Outer** | 0.0704 | 0 | 0 | 0 | 0.0001 | 0 | 0 | 0.0001 | 0 |
| **Outer** | 0.0685 | 0 | 0 | 0 | 0.0002 | 0 | 0 | 0 | 0 |
| **Outer** | 0.0004 | 0.0062 | 0.0017 | 0.0324 | 0.3371 | 0.0013 | 0.0009 | 0.0067 | 0.0008 |
| **Outer** | 0.0007 | 0.0072 | 0.0152 | 0.0543 | 0.3321 | 0.0099 | 0.0139 | 0.0448 | 0.0119 |
| **Halftide** | 0.001 | 0.0195 | 0.057 | 0.0172 | 0.0677 | 0.0109 | 0.0094 | 0.0272 | 0.0246 |
| **Halftide** | 0 | 0 | 0 | 0.0001 | 0.1528 | 0 | 0 | 0 | 0.0001 |
| **Halftide** | 0.0198 | 0.002 | 0.0182 | 0.0199 | 0.5451 | 0.0089 | 0.0294 | 0.2094 | 0.0294 |
| **Halftide** | 0 | 0.0169 | 0.0024 | 0.0118 | 0.0319 | 0.0002 | 0.0006 | 0.0298 | 0.2024 |
| **Halfway** | 0.0015 | 0.0002 | 0.0022 | 0.0119 | 0.0486 | 0.0006 | 0.0067 | 0.0678 | 0.0149 |
| **Halfway** | 0 | 0.0041 | 0.0008 | 0.0021 | 0.0135 | 0.003 | 0.0007 | 0.0342 | 0.0007 |
| **Halfway** | 0.0014 | 0.0062 | 0.0315 | 0.0452 | 0.4108 | 0.0199 | 0.009 | 0.1853 | 0.0082 |
| **Halfway** | 0.0001 | 0.0001 | 0.0035 | 0.0024 | 0.1747 | 0.0116 | 0.0001 | 0.0183 | 0.0197 |
| **Halfway** | 0 | 0.007 | 0.003 | 0.0033 | 0.0252 | 0.0017 | 0.0011 | 0.1738 | 0.0299 |
| **Humpy** | 0.0007 | 0.0001 | 0.0001 | 0.0105 | 0.025 | 0 | 0 | 0.0013 | 0.1993 |
| **Humpy** | 0.0007 | 0.0008 | 0 | 0.014 | 0.0134 | 0.0001 | 0.0003 | 0.0011 | 0.0448 |
| **Humpy** | 0.0048 | 0 | 0 | 0.0007 | 0.0138 | 0 | 0 | 0.0007 | 0.0091 |
| **Humpy** | 0 | 0 | 0 | 0 | 0.026 | 0 | 0 | 0.0006 | 0 |
| **Humpy** | 0.0006 | 0.0001 | 0 | 0.0029 | 0.0124 | 0 | 0 | 0.0037 | 0.2692 |
| **Humpy** | 0.0004 | 0.0049 | 0.0238 | 0.0279 | 0.1783 | 0.0017 | 0.001 | 0.0613 | 0.0503 |
| **Humpy** | 0.0004 | 0.2325 | 0.1366 | 0.0585 | 0.4175 | 0.1236 | 0.0006 | 0.1314 | 0.2067 |
| **Humpy** | 0.0001 | 0.002 | 0 | 0.0079 | 0.0457 | 0.0017 | 0.0001 | 0.0091 | 0.0003 |
| **Miall** | 0 | 0.0059 | 0.0015 | 0.0049 | 0.1732 | 0.0019 | 0.0166 | 0.048 | 0.0154 |
| **Miall** | 0.0005 | 0.3014 | 0.0202 | 0.2432 | 0.6357 | 0.0808 | 0.0011 | 0.1457 | 0.0343 |
| **Miall** | 0.0011 | 0.0356 | 0.0273 | 0.1293 | 0.0936 | 0.0043 | 0.0158 | 0.0148 | 0.0197 |
| **Miall** | 0.0007 | 0.3896 | 0.0646 | 0.2029 | 0.1546 | 0.0167 | 0.1664 | 0.4096 | 0.2006 |
| **Nth Keppel** | 0.0042 | 0.007 | 0.0575 | 0.4017 | 0.4213 | 0.1336 | 0.0285 | 0.1528 | 0.4669 |
| **Nth Keppel** | 0 | 0.0001 | 0.0008 | 0.0003 | 0.0271 | 0.0002 | 0 | 0.0369 | 0.0018 |
| **Nth Keppel** | 0.0007 | 0.0107 | 0.0161 | 0.1199 | 0.1372 | 0.0006 | 0.0006 | 0.056 | 0.0376 |
| **Nth Keppel** | 0.0001 | 0.0097 | 0.0229 | 0.095 | 0.2906 | 0.0049 | 0.0039 | 0.0125 | 0.0249 |
| **Passage** | 0.017 | 0.0118 | 0.0003 | 0.0024 | 0.0454 | 0.0011 | 0.0002 | 0.0412 | 0.0435 |
| **Passage** | 0.0006 | 0.0064 | 0 | 0.0587 | 0.0334 | 0.0017 | 0.0001 | 0.0037 | 0.1412 |
| **Passage** | 0.0386 | 0.026 | 0.1024 | 0.1605 | 0.3226 | 0.0777 | 0.0365 | 0.2527 | 0.11 |
