# Supplementary material for: A population genetic assessment of coral recovery on highly disturbed reefs of the Keppel Island archipelago in the southern Great Barrier Reef
Source: PeerJ. 2015 Jul 23;3:e1092. doi: 10.7717/peerj.1092 (PMC4517960; doi:10.7717/peerj.1092)

Fig. S1: Genetic diversity in A. millepora populations across the GBR: Difference between mean allelic evenness and allelic evenness at each site (A), and mean allelic evenness at each site (B).

A.

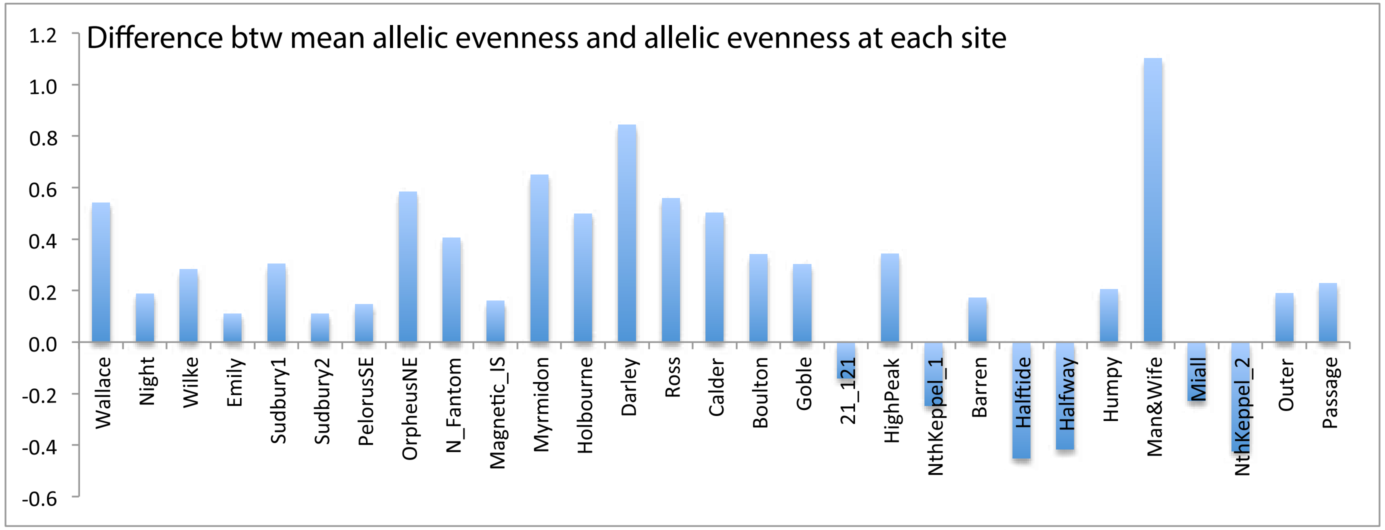

B.

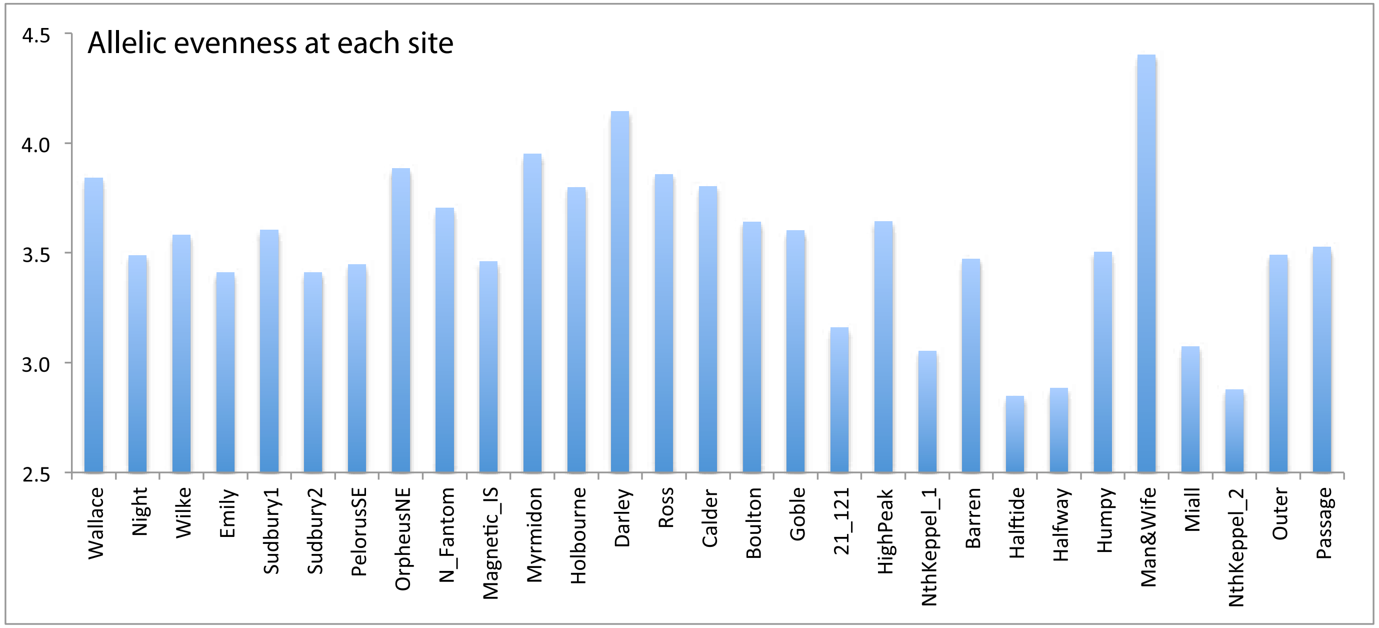

Supplement: Figure S1 [file peerj-03-1092-s006.pdf]
