# Supplementary figures and images for: A population genetic assessment of coral recovery on highly disturbed reefs of the Keppel Island archipelago in the southern Great Barrier Reef
Source: PeerJ. 2015 Jul 23;3:e1092. doi: 10.7717/peerj.1092 (PMC4517960; doi:10.7717/peerj.1092)

Fig S2: PCoA of pairwise  $D_{est}$  values of the Keppel Island populations.

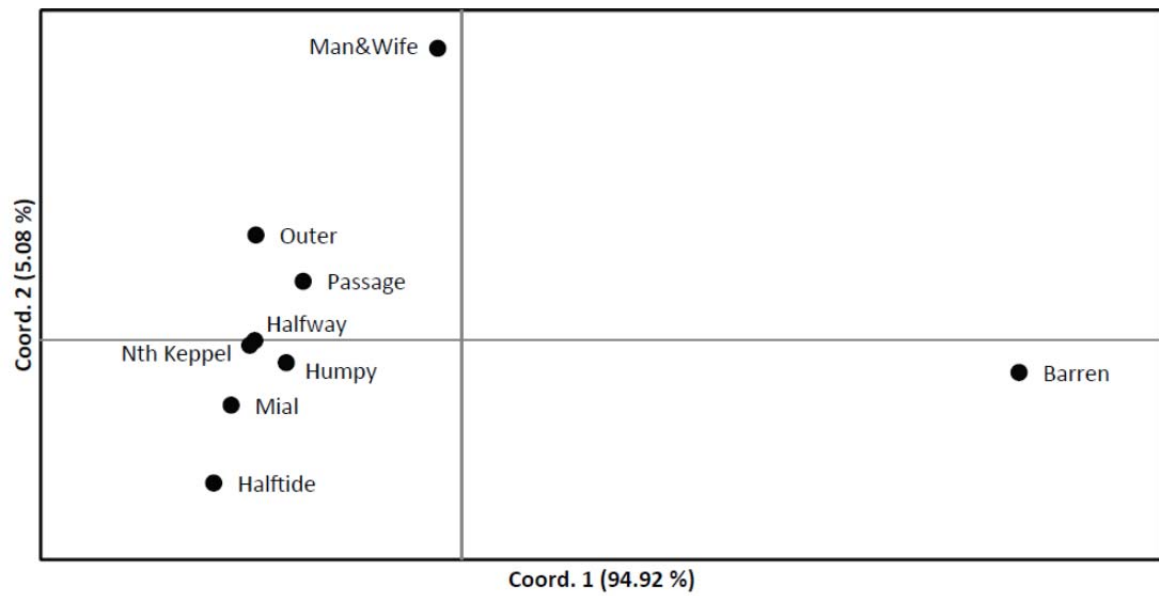

Supplement: Figure S2 [file peerj-03-1092-s007.pdf]

Fig S3: Isolation by distance analysis (IBD).

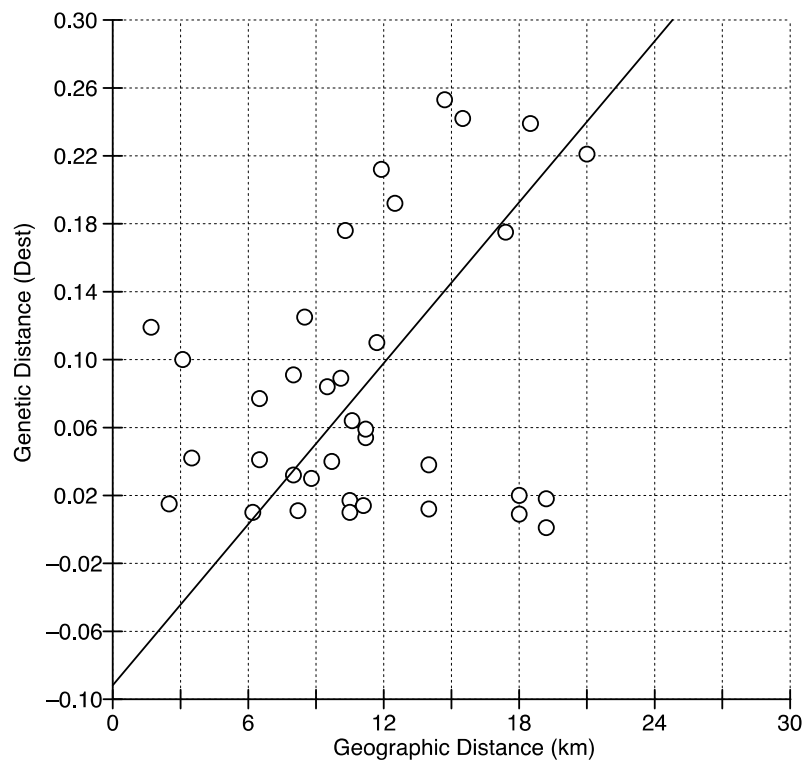

Supplement: Figure S3 [file peerj-03-1092-s008.pdf]

Fig S4: STRUCTURE LnProb(K) and Delta K and TESS DIC for Keppel Islands only.

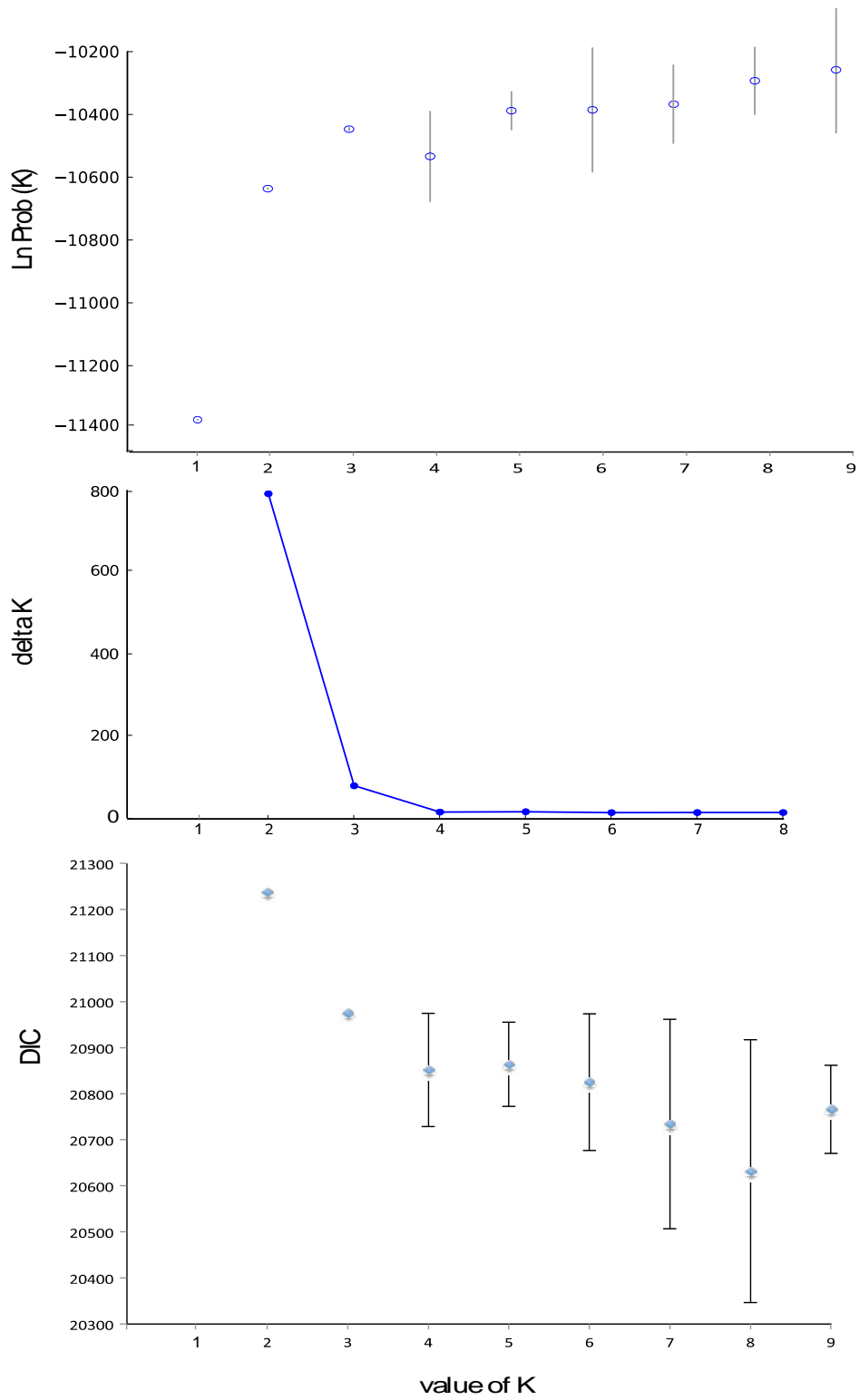

Supplement: Figure S4 [file peerj-03-1092-s009.pdf]

Fig S5: STRUCTURE (A & C) and TESS (B & D) results of Keppel Island populations for  $K = 2$  (A & B) and  $K = 3$  (C & D).

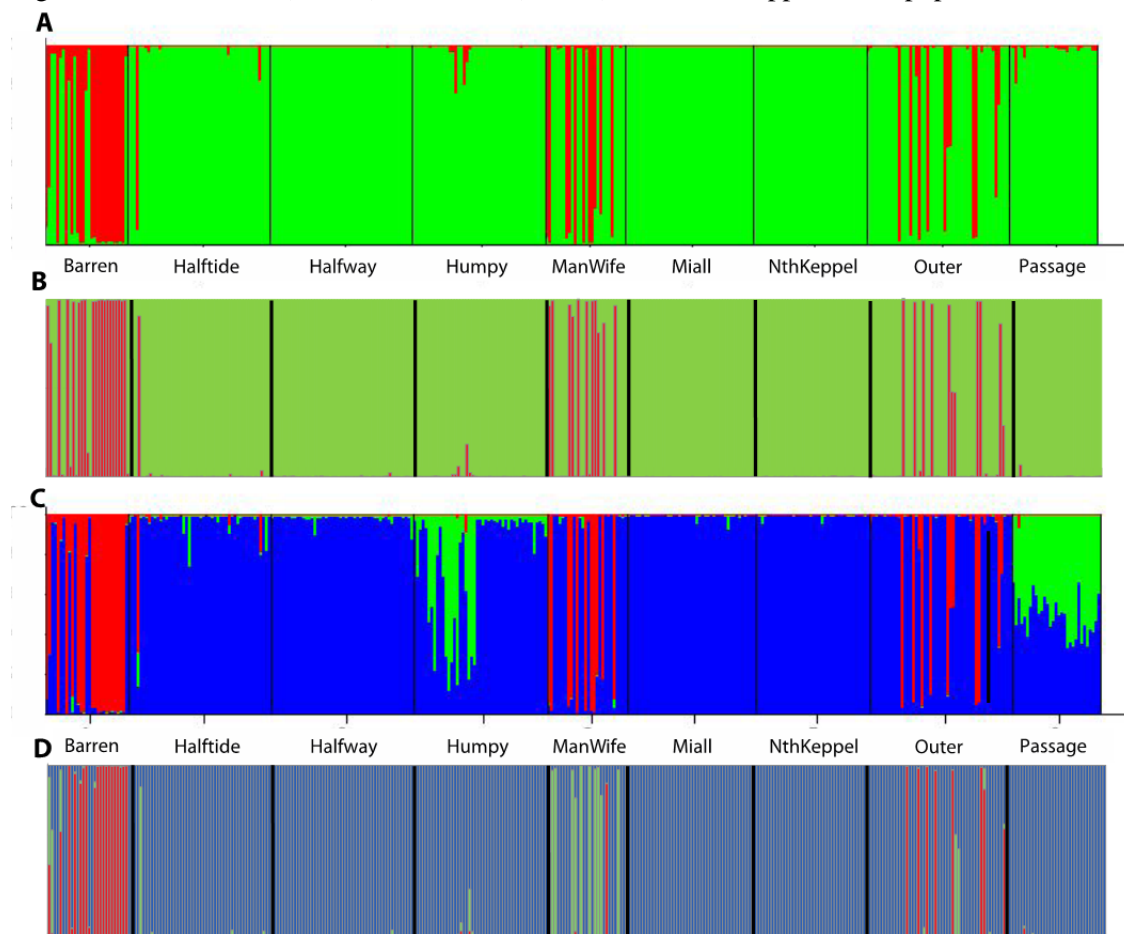

Supplement: Figure S5 [file peerj-03-1092-s010.pdf]

Fig S6: STRUCTURE LnProb(K) and Delta K and TESS DIC plot for combined data.

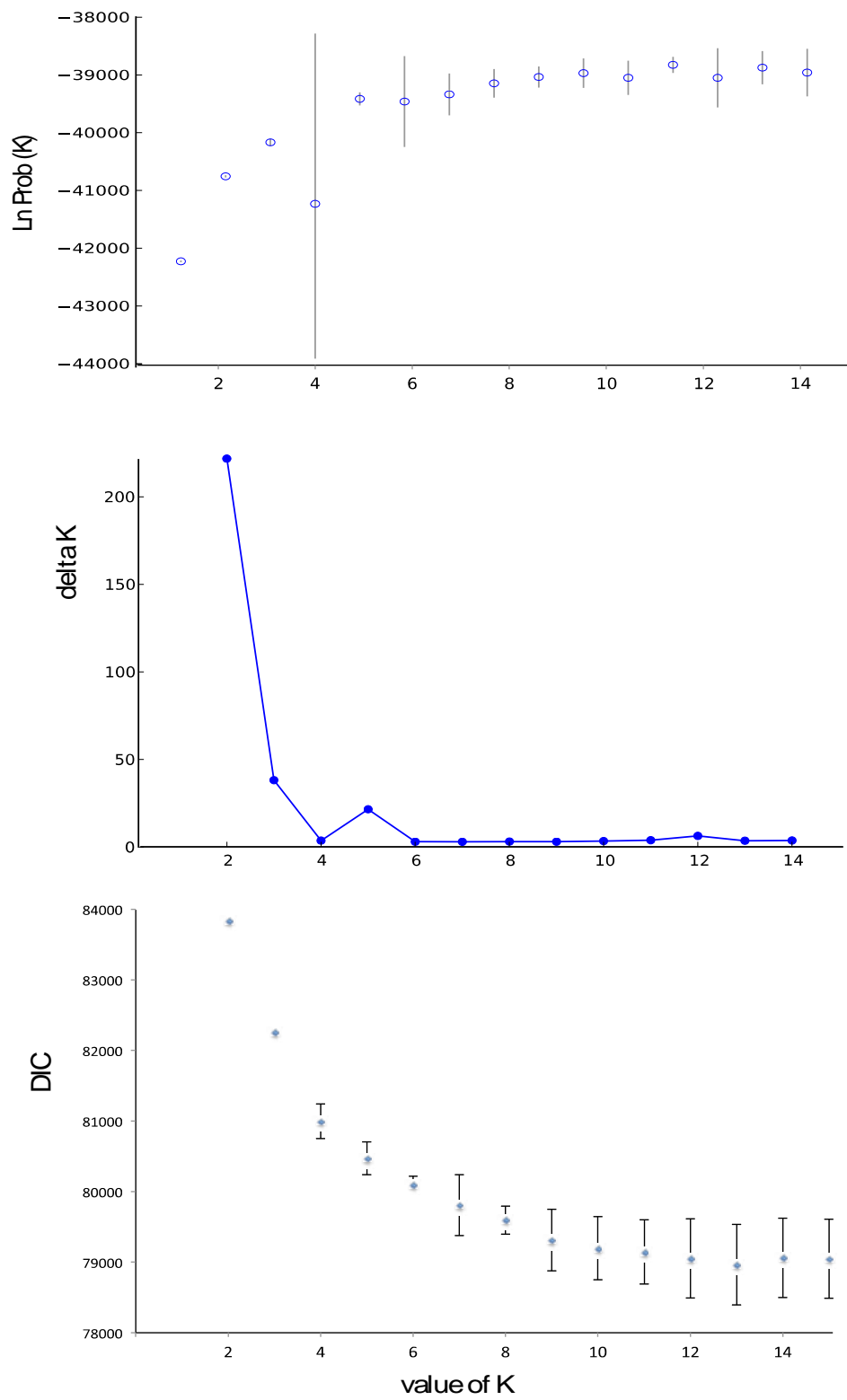

Supplement: Figure S6 [file peerj-03-1092-s011.pdf]
